# Attitudes about police and race in the United States 2020–2021: Mean-level trends and associations with political attitudes, psychiatric problems, and COVID-19 outcomes

**DOI:** 10.1371/journal.pone.0271954

**Published:** 2022-07-27

**Authors:** Catherine Vitro, D. Angus Clark, Carter Sherman, Mary M. Heitzeg, Brian M. Hicks

**Affiliations:** 1 Department of Criminology, University of Nebraska at Omaha, Omaha, Nebraska, United States of America; 2 Department of Psychiatry, University of Michigan, Ann Arbor, Michigan, United States of America; Manchester Metropolitan University, UNITED KINGDOM

## Abstract

The murder of George Floyd and subsequent mass protest movement in the summer of 2020 brought policing, race, and police brutality to the forefront of American political discourse. We examined mean-levels of attitudes about police and race using online surveys administered at five time points from June 2020 to October 2021 (*n* ~ 1000 at each wave) to adults living in the United States. There was a small increase in pro-police attitudes over this time (*d* = .24), and some evidence that mean-levels of pro-police attitudes increased more for Black participants (*d* = .51) than White participants (*d* = .20), and more for Democrats (*d* = .40) than Republicans (*d* = .15). Pro-police attitudes were much lower among Black participants than White participants (mean *d* = -1.04), and–relative to political independents–lower among Democrats (mean *d* = -.66) and higher among Republicans (mean *d* = .72). Pro-police attitudes had large associations with a variety of conservative or right-wing political attitudes (e.g., approval of Donald Trump) and COVID-19 variables (e.g., disapproval of government mandates and restrictions), but were unrelated to psychiatric problems and substance use. These results validate a new measure of police attitudes, provide information on trends in police attitudes over the 15 months following the largest mass protests against police brutality in American history, and begin to establish the nomological network of police attitudes, finding that pro-police attitudes are firmly within the right-wing coalition of American politics.

## Introduction

In the United States, there is a tradition of valorizing the role of police officers in society. Special honors are bestowed at the funerals for police officers killed in the line of duty and monuments to these honored dead have been erected in cities throughout the United States. Local police departments often hold award ceremonies to recognize acts of bravery in the force [1]. Nationally, the Public Safety Officer Medal of Valor–comparable to the military’s Medal of Honor–was established by former President Clinton in 2000 and is awarded by the president to public safety officers for acts of bravery. Since 1993, an average of 55% of Americans state that they have “a great deal or quite a lot of confidence” in the police, although this varies considerably when comparing White and non-White populations [2]. Further, the police consistently rank in the top three for confidence among major U.S. societal institutions [2, 3].

Despite the perception that policing is an especially dangerous and heroic job, hence meriting its valorization, police officers rank 22^nd^ in on the job deaths (11 fatalities per 100,000 workers), behind such occupations as fishing and hunting workers (145 deaths per 100,000 workers), loggers (69 deaths per 100,000 workers), aircraft pilots and flight engineers (62 deaths per 100,000 workers), garbage collectors (35 deaths per 100,000 workers), delivery drivers (27 deaths per 100,000 workers), and structural iron and steel workers (26 deaths per 100,000 workers) [4]. Police officers also have higher levels of compensation with a median annual salary of $67,290 [5], relative to $42,350 for loggers [6] and $53,210 for structural iron and steel workers [7], despite all three typically having an entry-level education requirement of a high school diploma or equivalent [5, 7]. Police officers are considered more valuable, more worthy of accolades, and more heroic than other occupations, despite working in a less dangerous occupation.

### Portrayals of police in popular culture

One reason for these perceptions is the portrayal of police officers in television and movies. Americans are more likely to watch television than engage in any other activity other than sleeping and working [8, 9], and social science research has shown the significant influence that television can have on different aspects of society such as body image and eating behavior [10] or racial prejudice [11, 12]. During the 2000s, shows revolving around law enforcement and crime have made up a large proportion of available television. In the fall of 2019, over 60% of prime-time dramas on the four main U.S. broadcast networks were series focused on crime, and crime shows made up nearly 20% of all scripted shows on broadcast networks, down from almost 29% in 2014–2015 [13, 14]. Some crime shows such as *Brooklyn Nine-Nine* and *The Wire* have attempted to critique the policing profession in their portrayals, but even these shows have been criticized as being pro-cop as police officers are still protagonists of the series and the individual characters themselves are often likable [15, 16]. The positive portrayals of police officers in media does not always reflect the reality of the policing profession, an idea that has become known as “copaganda” [17].

### Demographic correlates of attitudes about police

Positive perceptions of police have never been universal, however. Non-White race, lower social class, residing in lower income and higher crime neighborhoods, and younger age are all associated with less favorable attitudes about police [18–21]. The largest racial gap in attitudes is between Black and White Americans. Since 1993, the percentage of Black Americans with “a great deal or quite a lot” of confidence in the police is typically 25-percentage points lower than White Americans [22]. Race has a strong association with lower income and education and is associated with a greater likelihood of residing in poor neighborhoods [23]. Some research suggests that socioeconomic status is more important than race in determining attitudes about police, or that racial differences tend to disappear after accounting for perceptions of neighborhood disorder or social class [21]. Given the strong relationships among race, class, and neighborhood residence in the United States, however, it is important to be cautious when making inferences regarding their unique influences after adjusting for their overlap.

Personal experiences with police tend to be one of the strongest predictors of attitudes about police [24, 25], and may mediate the relationship between demographic variables and perceptions of the police. Formal contact with the police, whether through personal victimization or police-initiated contact, is associated with more negative perceptions of officers [19]. In contrast, informal contact with the police, which still occurs in an official capacity but is not associated with criminal activity, is associated with more positive perceptions of officers [19].

The amount of formal or informal contact people have with police is also related to race, neighborhood, socioeconomic status, and age, with younger racial minorities in lower-income areas reporting higher levels of formal contact with police officers [26–29]. One of the reasons for this is that more intensive policing tends to be concentrated in small geographic areas with high crime rates that are typically urban. This use of “hot spot policing” follows research indicating that it is effective for policing resources to be concentrated in areas with more crime [30]. However, these tactics may also lead to increased resentment and negative perceptions of officers due to greater formal contact [30].

It is important to consider these factors in conjunction with the view of police in the media. If a person’s only contact with police is through fictional shows, social science research indicates these shows help shape people’s views of the policing profession [31]. Regular viewing of crime-based reality programs significantly can increase confidence in police, but these effects are only observed among White participants and do not impact victims and individuals with personal arrest experience [31]. For example, ratings data in 2017 showed that *NCIS* was the most popular show among White viewers but did not rank in the top 5 for any other racial group [32].

### Political correlates of attitudes about police

Prior to the 2000s, limited research was done regarding how political ideology is associated with perceptions about the police [33]. While there have been notable instances of political alignment between conservative political parties and law enforcement (e.g., Richard Nixon’s call for “law and order” in the 1968 presidential election [34, 35]), 20th century research on differences in perceptions of police focused on demographic characteristics rather than political ideology. Of the few studies that looked at political party and policing perceptions in the United States prior to 2000, one found that Republicans held more favorable attitudes toward law enforcement than Democrats [36], and another found no significant difference between Democrats and non-Democrats [37]. Also, a study in Canada found that conservatism had a strong positive correlation with attitudes towards police [38].

In the last decade, the divide between political affiliation and attitudes towards police has come to the forefront, particularly following highly publicized events of police brutality [39–41]. Republicans report higher confidence in the police [31, 42], rate the police higher in measures of honesty and ethics [43], and have a more favorable attitude towards the police in general than Democrats [18]. These political divisions are also apparent when viewing police misconduct toward racial and ethnic minorities. For example, a Pew Research Center poll found that 61% of Republicans compared to 21% of Democrats endorsed the belief that race was “getting too much attention” following the shooting of Michael Brown, an unarmed 18-year-old Black man who was fatally shot by a police officer in Ferguson, Missouri in 2014 [41]. Furthermore, Democrats are more likely than Republicans to believe major changes are needed to police in the United States [44].

The ties between law enforcement and the Republican party in the United States have grown stronger since the ascendance of Donald Trump to the presidency, especially as policing became a central issue in Trump’s 2020 reelection campaign [45]. Trump called himself the “law-and-order candidate” during his 2016 election campaign [46], and the “president of law and order” during his reelection campaign in 2020 [47]. Trump’s candidacy was backed by endorsements from local and national police unions such as the Fraternal Order of Police, the largest national union group, as well as unions representing federal law enforcement agents such as the U.S. Border Patrol and Immigration and Customs Enforcement (ICE) [45, 48, 49]. Trump maintained this position during the George Floyd protests in the Summer of 2020, tweeting “LAW & ORDER, NOT DEFUND AND ABOLISH THE POLICE. The Radical Left Democrats have gone Crazy!” amidst calls for police reform and defunding the police [50]. Soon, “Thin Blue Line” flags were prominently displayed during Trump reelection rallies during the fall of 2020 [51], which had previously merely indicated support for police officers but have come to signal opposition to racial justice movements and have been adopted by White supremacists [52–54]. Our own research has found that since 2020, pro-police attitudes are strongly associated with approval of Trump, affiliation with the Republican party, and other conservative and ring-wing attitudes [55].

### Highly publicized events of police misconduct and attitudes about police

Within the background of the demographic correlates and relatively positive mean attitudes about police in the United States, highly publicized events of police misconduct and brutality can have a significant negative impact on these attitudes, but these effects are usually not long-lasting [56, 57]. Civil unrest due to cases of police brutality have been prevalent throughout American history, and the resulting impact on public perception has been similar. Polling data often shows a significant downward trend of confidence in local or national police forces following an incident of police brutality, but this tends to recover to pre-event levels in the months and years after the incident [22, 56–59]. For example, following the beating of Rodney King (March 3, 1991) and subsequent acquittal of the police officers involved in the incident (April 29, 1992) that incited the 1992 Los Angeles riots, Los Angeles Times’ polls showed support for the LAPD plummeted before increasing and eventually returning to pre-incident levels over the next few years [57]. After waves of civil unrest in Ferguson and highly publicized police killings of multiple Black men in 2014–2015, Gallup polls recorded the lowest levels of police confidence since 1993 [40]. From 2015 to 2017, however, Gallup polls recorded that confidence in police rebounded to its high historical average [58]. While the declines in confidence in police were more pronounced among Black than White Americans, the trends remained the same: police brutality incidents have adverse effects on public perceptions of police, but these effects often disappear over time [40, 57, 58, 60].

In 2013, the #BlackLivesMatter (BLM) hashtag was started following the acquittal of a neighborhood watch captain who killed Trayvon Martin, a 17-year-old from Florida [61, 62]. BLM was propelled by the broader use of social media to publicize instances of police brutality, which increased awareness globally. Between 2013 and 2018, the hashtag was used almost 30 million times on Twitter [63]. BLM became a decentralized global movement with a network of more than 40 chapters worldwide [61]. The dissemination of the video documenting the murder of George Floyd by a police officer in Minneapolis on social and broadcast media spurred what became the largest mass demonstrations in American history in the summer of 2020 [64]. The BLM movement took center stage in American public discourse and reached its highest levels of public support, though it was met by counter reactionary responses (e.g., #BlueLivesMatter, a pro-police movement that argues for protection of law enforcement under hate crime statutes [65–67]), and its support declined due to instances of confrontations with police and rioting during the summer protests, and its unpopular proposal to defund police departments.

Each of these, and many other, instances of police brutality against Black individuals have been followed by significant racial divide. Race has become important in these conversations following the highly publicized mistreatment of Black individuals by law enforcement, and thus the relationship has been viewed as contentious and biased against Black people [68]. However, this focus on the racialized nature of police misconduct has also been divided across racial lines. Following the fatal shooting of Michael Brown, for example, a New York Times poll found that 58% of White adults felt race does not affect police use of deadly force, but only 20% of Black adults felt the same [69]. As these events of police misconduct and police brutality continue to be prevalent throughout American history, and primarily occur toward Black individuals, the more race comes to the forefront especially for Black communities. Thus, Black individuals typically have more negative reactions in their attitudes toward police following highly publicized events of police misconduct.

### Current aims

We examined whether this trend remained consistent in 2020 and 2021, despite the larger scale of the George Floyd protests. These protests coincided with the lowest recorded confidence in police, the highest level of support for the BLM movement, and the most extreme calls for police reform in American history including defunding and abolition [70–72], suggesting there may have been a longer-lasting shift in public opinion regarding attitudes about police.

Initial reports of police confidence in the United States over a year after the George Floyd protests, however, seem to follow the expected trend. In 2019, people endorsing high confidence in the police was at 53%, which dropped to 48% in 2020 and rose again to 51% by 2021 [22, 71]. Similarly, in the summer of 2020, 60% of Americans trusted the BLM movement “to promote justice and equal treatment of the people” compared to 56% of respondents who trusted police to do the same. By the summer of 2021, however, trust in the BLM movement fell to 50% and trust in the police increased to 69% [73].

We examined these ideas in more depth using online surveys completed by adults living in the United States on five occasions from June 2020 to October 2021. We built upon prior studies in several ways. First, rather than a poll question approach, we took a psychometric perspective in conceptualizing police attitudes as a trait construct that can be measured using the commonality of responses to a variety of items assessing different aspects of police attitudes. This approach provides a more reliable and precise estimate of individual differences in police attitudes than a single question. Second, we examined mean-level change and rank-order stability in police attitudes in the 15 months following the George Floyd protests in the summer of 2020.

Finally, we examined the associations between police attitudes and a larger array of variables than most prior studies. This included demographic variables (age, sex, race, education, income) and several variables related to political attitudes. The period of 2020–2021 coincided with the beginning of the COVID-19 pandemic and other major social and political disruptions in the United States. Consequently, we also assessed attitudes about contemporary political events and actors such as approval of former President Trump and current President Biden as well as attitudes about controversies about the 2020 presidential election (the Big Lie) and the Insurrection of January 6, 2021. Additionally, we assessed several variables related to the COVID-19 pandemic including attitudes about government restrictions, frequency of engaging in safety behaviors (e.g., mask wearing), worries about COVID-19, and anti-vax attitudes. Lastly, we examined associations between personality traits including the Big Five traits and Right-Wing Authoritarianism (conservative political and social attitudes) and mental health problems and substance use, which have received little attention in terms of potential associations with police attitudes. For example, certain traits and behaviors like aggression, impulsivity, antisocial behavior, violence, heavy alcohol and drug use are associated with illegal activities and increase the likelihood of arrest and formal contact with police. Consequently, these traits and behaviors are likely to be associated with more negative attitudes toward police.

We predicted scores on our measure of pro-police attitudes would increase from June 2020 to October 2021. We also predicted lower mean-levels of pro-police attitudes for non-White (especially Black) participants relative to White participants. Relative to independent or politically unaffiliated participants, we predicted that Republicans would endorse higher and Democrats lower mean-levels of pro-police attitudes, respectively. Additionally, we predicted that pro-police attitudes would be consistently associated with more conservative or right-wing political attitudes (e.g., approval of Donald Trump). Further, because the COVID-19 pandemic has been so politicized in the United States, we predicted pro-police attitudes would be associated with greater skepticism about the seriousness of COVID-19, less approval of government restrictions, and less engagement in safety behaviors. Finally, we predicted that antisocial behavior, violence, and substance use would be associated with lower pro-police attitudes.

## Methods

### Sample ascertainment

Data were collected at five different time points: June 9 through June 22, 2020; September 24 through October 25, 2020; January 14 through February 25, 2021; May 17 through June 28, 2021; and September 16 through October 11, 2021. Recruitment entailed an actively managed, double-opt-in research panel with Qualtrics XM survey software. Recruitment was designed to ascertain a sample similar to the United States general population using quotas for the demographic variables of age, sex, and race that were monitored while the survey was in field. Respondents were recruited using a dashboard-style web page on the Qualtrics website and cellphone app where participants see a list of surveys that they have the option to participate in. Recruitment was also conducted through emails sent to established panel members within the Qualtrics database. In all recruitment methods, potential participants received information on the estimated length of the survey and compensation for completing it. Specific details about the survey content were not available until the participants opted-in to avoid self-selection bias. Upon opting into the study, participants read and provided an electronic signature on a consent form containing an overview of the survey contents. Participation was voluntary and anonymous as no individually identifying information was collected. Contact information for the research team was provided in case participants had questions about the survey. The University of Michigan Medical School Institutional Review Board (IRB) reviewed all study protocols. After participating in a survey wave, attempts were made to recontact respondents to participate in all subsequent wave(s). Following wave 1, new respondents were allowed to join the study once participation by prior respondents plateaued to maintain a consistent sample size (~1000 participants) and quotas for the specified demographic variables across waves.

After the surveys were closed, the data was manually checked and a small number of respondents (6 in wave 1, 20 in wave 2, 13 in wave 3, 27 in wave 4, 12 in wave 5) were excluded due to inconsistent and illogical responses. Single measures were also excluded on a case-by-case basis if all other responses from that participant were within a plausible range of values. The final sample sizes were 1008, 1004, 1005, 1019, and 1038 participants for waves 1 through 5, respectively, with 2809 unique participants. For the number of assessments completed, 1718 participants completed one wave, 421 completed two waves, and 670 completed three or more waves. The median response time for completing the surveys ranged from 22.6 to 28.1 minutes.

Table 1 reports the demographic characteristics of the total sample of unique participants collapsed across waves. The demographic characteristics of the total sample was similar to those reported by the U.S. Census Bureau for the United States population for 2019 for race and household income. Female participants, however, constituted a higher proportion of the sample than the U.S. population (53.7% vs. 51.7%). The sample’s mean educational attainment was higher than the U.S. general population due to lower rates of people with a high school diploma or less (18.6% sample vs. 41.0% U.S. general population) and higher rates of people with a Bachelor’s (31.6% sample vs. 19.0%) and graduate degrees (22.2% sample vs. 11.0%). The mean age of the sample was younger than the U.S. population due to higher representation of people in the age range 25–44 (43.1% sample vs. 34.4%) and lower representation of people ages 45 and above (44.0% sample vs. 52.9%). In terms of household income, people with lower incomes (< $50,000) were comparably represented (36.9% sample vs 37.8%), people with middle incomes ($50,000 to $100,000) overrepresented (32.8% sample vs 28.6%), and people with higher incomes (> $100,000) slightly underrepresented (30.4% sample vs 33.6%). In terms of political party affiliation, people affiliated with the Democratic party (41.8% sample vs 33.0%) were overrepresented while people affiliated with the Republican party (25.0% sample vs 29.0%) and people who identified as independent or unaffiliated (28.4% sample vs 34.0%) were underrepresented relative to the 2020 general electorate [74].

**Table 1:** Descriptive statistics for total sample across waves.

	Total (*N* = 2809)
	% (*n)*
**Sex**	
Male	46.3 (1297)
Female	53.7 (1502)
**Age, years**	
Mean (SD)	43.9 (16.2)
**Education**	
High school diploma or less	18.6 (516)
Some college	27.7 (769)
Bachelor’s degree	31.6 (879)
Graduate degree	22.2 (617)
**Annual Household Income**	
Less than $50,000	36.9 (1025)
$50,000-$99,999	32.8 (911)
$100,000 +	30.4 (845)
**Political Affiliation**	
Democratic	41.8 (1153)
Republican	25.0 (690)
Independent/Not Registered	28.4 (784)
Third Party	4.7 (129)

**Note**. Percentages refer to valid responses. Rates of missing data were as follows: sex, 0.4% (*n* = 10); age, 0.4% (*n* = 11); race, 0.5% (*n* = 14); Hispanic ethnicity, education, and income, 1.0% (*n* = 28); political party, 1.9% (*n* = 53).

## Measures

### Attitudes about police and race

Beginning at wave 1, we wrote and included items to assess attitudes about protests against police brutality (6-point response option format from *strongly suppor*t to *strongly oppose*), police in general (*positive*, *mostly positive*, *mostly negative*, *negative*), and police misconduct and racial bias in policing in the United States (6-point response option format from *strongly approve to strongly disapprove*). Five items were assessed at each wave; two items were dropped after wave 2 due to lack of relevance and weaker psychometric properties relative to the other items; an item specific to support for BLM was added at wave 2; additional exploratory items were also included in waves 4 and 5. Items are listed in Table 2 and further details on the psychometric properties of the items and scale are provided in the Results section.

**Table 2:** Items assessing attitudes about police and standardized factor loadings for each wave.

Item	W1	W2	W3	W4	W5
To what extent do you support recent protests against police brutality in the U.S.	-.82	-.81			
How would you describe your views of the police in general	.77	.77	.73	.72	.69
The police treat black and white people equally.	.85	.84	.80	.79	.75
Police misconduct is mostly due to “a few bad apples”.	.66	.65			
Police are more concerned about exerting their authority than protecting citizens and enforcing laws.	-.77	-.76	-.72	-.72	-.68
Police misconduct is mostly due to institutional forces such as racism and the “code of silence” among police officers.	-.77	-.76	-.73	-.72	-.68
A black person is more likely to be arrested or treated more harshly than a white person for committing or being suspected of committing the same crime.	-.87	-.86	-.82	-.81	-.77
To what extent do you support the “Black Lives Matter” movement.		-.92	-.87	-.86	-.82
Police officers are true heroes.				.76	.72
I admire the bravery of police officers.				.74	
Police should be prosecuted for killing people when they are not in immediate danger, even if it was a mistake (e.g., manslaughter).				-.66	-.63
If people just do what police tell them to, they won’t get hurt.				.74	.70
Factor Reliability					
ω	.92	.93	.90	.90	.89
*H*	.93	.94	.92	.91	.90
Factor Mean	.00	.17	.13	.20	.23
Factor Variance	1.00	.99	.89	.87	.79

Note. ω = coefficient omega; *H* = *H* index of factor reliability. Results are from a model with factor loadings for the same item constrained across waves (W). Model fit was: χ^2^ = 9393.97, *df* = 882, *p* < .001; RMSEA = .059; SRMR = .094; CFI = .950; TLI = .956.

### Time-invariant predictors

#### Demographics

Demographic characteristics included age, sex assigned at birth (male, female), race (White, Black, other non-White), Hispanic ethnicity (Yes, No), annual household income, and educational attainment. Political party (Democrat, Republican, and Independent or unaffiliated) was also included as a predictor of police attitudes.

#### Personality

The Big Five Inventory-2 short form (BFI-2-S; 30-items) was used to assess extraversion (sociability, assertiveness, energy level; α = .71), agreeableness (compassion, respectfulness, trust; α = .76), conscientiousness (organization, productiveness, responsibility, α = .78), negative emotionality (anxiety, depression, emotional volatility, α = .85), and open-mindedness (aesthetic sensitivity, intellectual curiosity, creative imagination, α = .68).

The Right-Wing Authoritarianism (RWA; 22-items) scale was included in the wave 2 assessment and used to assess liberal (e.g., *Everyone should have their own lifestyle*, *religious beliefs*, *and sexual preferences*, *even if it makes them different from everyone else*.) and conservative (e.g., *The only way our country can get through the crisis ahead is to get back to our traditional values*, *put some tough leaders in power*, *and silence the troublemakers spreading bad ideas*.) political and social attitudes (α = .88 [75]). RWA scores were coded so that high scores indicated agreement with conservative attitudes and disagreement with liberal attitudes.

### Time-varying predictors

#### Political attitudes

*Attitudes about race and immigration in the United States*. We used a six-item scale with items modeled on a 2019 Pew Research Center poll to assess attitudes about race (e.g., *When it comes to giving Black people equal rights with Whites*, *our country has*: *(1) Not gone far enough*, *(2) Been about right*, *(3) Gone too far*.) and immigration (e.g., *If America is too open to people from all over the world*, *we risk losing our identity as a nation*.) (In a Politically Polarized Era, Sharp Divides in Both Partisan Coalitions, 2019) (α = .89 [55]).

#### Presidential approval

We asked respondents to report their approval of President Biden’s job performance (waves 4 and 5) in general and past President Trump’s overall approval (waves 1 thru 3) *(strongly approve*, *approve*, *somewhat approve*, *somewhat disapprove*, *disapprove*, *strongly disapprove)*.

#### Media bias

We used a six-item scale that asked respondents to report their agreement with statements about partisan biases in news sources either to a specific news source (e.g., *CNN is fake news*.) or news bias in general (e.g., *The mainstream media is biased against conservative viewpoints*.) (α = .92; [55]). Items were coded so that higher scores indicate a belief in a bias against conservative or right-wing political attitudes and politicians.

#### Pro-gun attitudes

We used a four-item scale to assess attitudes about state regulations on the purchase and ownership of personal firearms and attitudes about gun ownership in general (e.g., *The best defense against a tyrannical government is a well-armed citizenry)* (α = .84; [55]).

#### Pro-insurrection attitudes

We used a five-item scale that asked respondents to report their agreement with statements about the attempted insurrection of the Capitol on January 6, 2021 (e.g., *The Pro-Trump protesters are patriots who are trying to protect our democracy*) (α = .84).

#### Rigged 2020 presidential election

We used a seven-item scale to assess perceptions of the 2020 presidential election results (e.g., *Democrats cheated their way into winning the 2020 election*.) (α = .86).

#### Trump idolatry

We used a four-item scale to assess reverence for former President Donald Trump (e.g., *Only President Trump is capable of solving the nation’s problems*.) (α = .77).

#### Psychiatric problems

*Mental health problems*. For mental health problems, we used the general Depression (20-items; α = .95) and Suicidality (6-items; α = .93) scales from the Inventory of Depression and Anxiety Symptoms (IDAS; [76]) and the Generalized Anxiety Disorder-7 (GAD-7 [77]) (α = .94) scale to assess suicidal thoughts, self-harm harm behaviors, and overall depressive and non-specific anxiety symptoms over the past two weeks.

#### Substance use

We assessed alcohol use by calculating the mean of three items related to drinking alcohol in the past 30 days including average number of drinks per week, number of binge drinking episodes (i.e., five or more drinks on one occasion), and greatest number of drinks consumed in a 24-hour period (α = .87). We also assessed nicotine use using frequency of smoking cigarettes, using smokeless tobacco, or e-cigarettes (*Never*, *once or twice*, *occasionally but not regularly*, *regularly in the past*, *regularly now*). Response options *“regularly in the past”* and *“regularly now”* were coded the same for the analyses. The highest frequency reported among the three nicotine questions was used for the nicotine use variable.

#### COVID-19 attitudes and behaviors

*Anti-vax beliefs*. We used a four-item scale assessing general support for vaccinations to assess anti-vaccination (i.e., anti-vax) attitudes (e.g., *Vaccines are more dangerous than the diseases they are trying to prevent*.) (α = .91 [78]).

#### COVID-19-related safety behaviors

Participants were asked how often they followed the “social distancing”, or “shelter-in-place” restrictions put in place in your community in the past three months, and how often they wear a mask in public, both indoors and outdoors (*never*, *seldom*, *sometimes*, *often*, *always*).

#### Attitudes about COVID-19-related government mandates and restrictions

Participants were also asked three questions to assess attitudes about government responses to COVID-19 including the necessity of COVID-19 related restrictions (*very necessary* to *very unnecessary*), approval of their state government’s social distancing restrictions (*strongly approve* to *strongly disapprove*), and attitudes about the pace at which their community was lifting social distancing restrictions (*much too soon* to *much too late*) (α = .83).

#### COVID-19 risk

We used a six-item scale to assess perceptions of risk for contracting or spreading the COVID-19 virus while engaging in common activities including dining in a restaurant indoors and outdoors, going to a bar indoors and outdoors, attending a large gathering indoors (more than 15 people) and outdoors (more than 25 people) (α = .92).

#### COVID-19 skepticism

We a four-item scale that asked participants to rate their level of agreement with statements about the severity COVID-19 and the public health consequences of the pandemic (e.g., *COVID-19 is no worse than the flu)* (α = .91; [55]).

#### COVID-19 worry

We used 4-items from the COVID-19 Adolescent Symptom and Psychological Experience Questionnaire (CASPE) to assess COVID-19 related negative emotions during the past 30 days. Participants reported their level of stress related to the uncertainty of the future because of the COVID-19 pandemic and their level of worry of infection for themselves and their friends and family, and that their physical health would be impacted by COVID-19 (α = .92).

### Data analytic strategy

First, we evaluated the psychometric properties of the police items as measures of an overall police attitude construct. Because some items were not administered at each wave, we needed a model that allowed for missing data, but still maximized the available information so that police attitude scale scores were on the same metric and so could be validly compared across waves. To do so, we fit a five-factor categorical confirmatory factor analytic measurement model fit using mean and variance adjusted weighted least squares estimation (WLSMV) [79]. A single factor was modeled to account for the covariance among police items administered in the same wave, and items were only specified to load on the factor for the assessment in which they were administered. Items were keyed so that higher scores were associated with more “pro” or less critical attitudes about police.

Next, we examined change and stability on the factor scores of our measure of police attitudes from Summer 2020 to Autumn 2021. Initially, we fit a latent growth curve with structured residuals (LGC-SR) model [80] but found that there was no variability in the slope or rate of change parameter even though there was mean-level change over time. This was likely due to two factors: (1) most participants provided only one or two assessments which limited statistical power to detect within-person change, and (2) the interval between assessments was relatively short (3 months) which contributed to high rank-order stability on the police attitudes measure. While we were still able to describe mean-level changes over time, this limited our ability to account for individual differences in within-person change. Therefore, most of our analyses focused on examining correlates of police attitudes at the between-person level with variants of the random intercept model.

**Fig 1:**
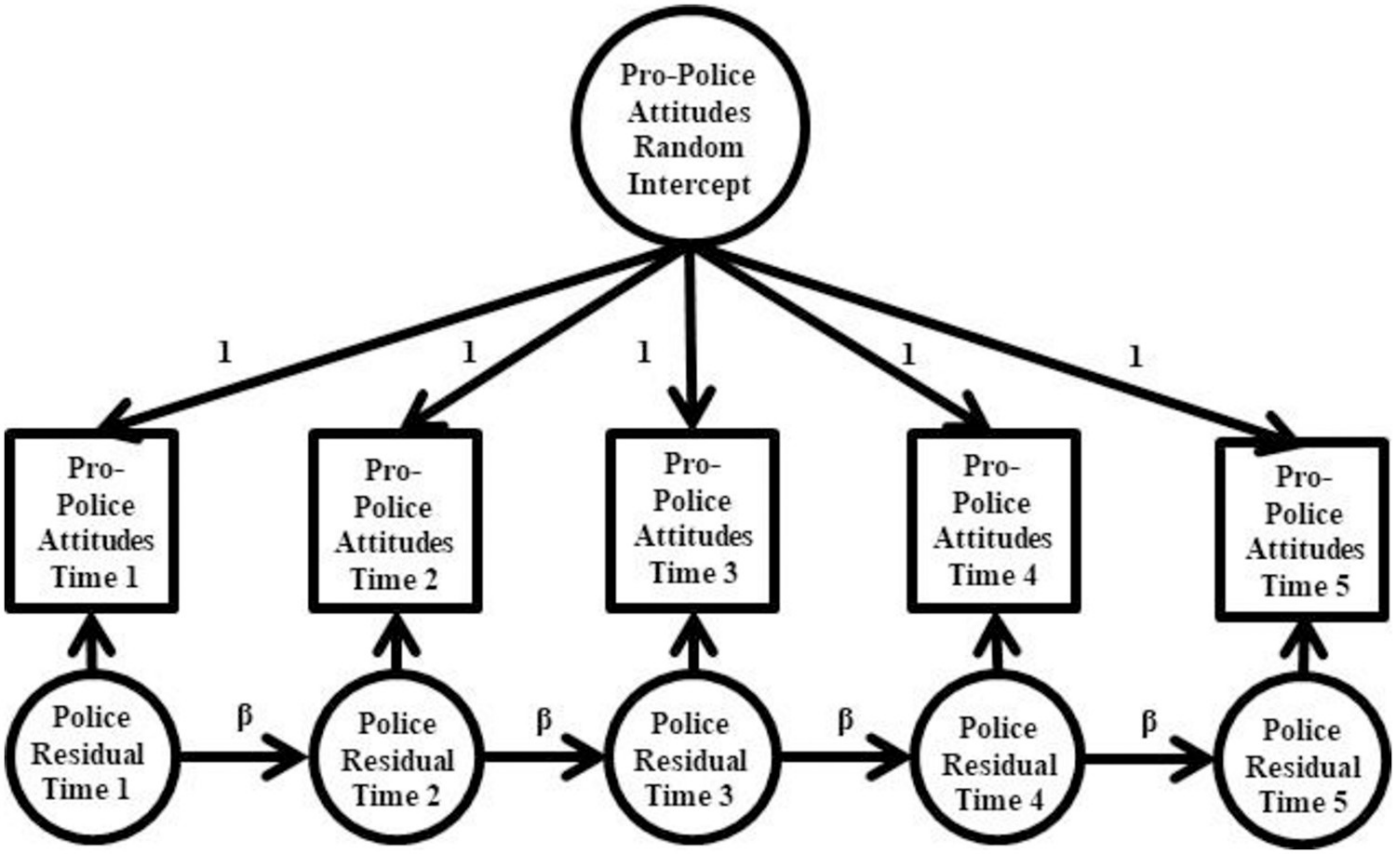
Random Intercept Autoregressive Model (RIAM). β = autoregressive path. Variances and mean structure omitted from figure for clarity of presentation. All factor loadings were fixed to 1. All autoregressive paths were freely estimated.

First, we fit a univariate Random Intercept Autoregressive Model (RIAM; Fig 1) to estimate pro-police factor scores over time. In this model, factor scores of police attitudes at each wave were specified to load on a random intercept factor with factor loadings fixed to 1 and factor score intercepts freely estimated across time to preserve mean change across time. The random intercept factor then measures the extent to which a person’s pro-police attitude scores were consistently higher or lower than the sample mean over time. The random intercept mean was fixed to 0, and the random intercept variance was freely estimated. Each factor score was also specified to load on a time-point specific residual factor with factor loadings fixed to 1, the residual factor means fixed to 0, and the residual factor variances freely estimated. Autoregressive paths were specified running from one residual factor to the subsequent residual factor (β in Fig 1). After fitting the RIAM, we examined the associations between a series of time-invariant covariates and the pro-police random intercept factor. In these models, the random intercept factor was regressed on several covariates simultaneously including demographic variables, personality traits, and political party affiliation to estimate their unique associations with police attitudes.

**Fig 2:**
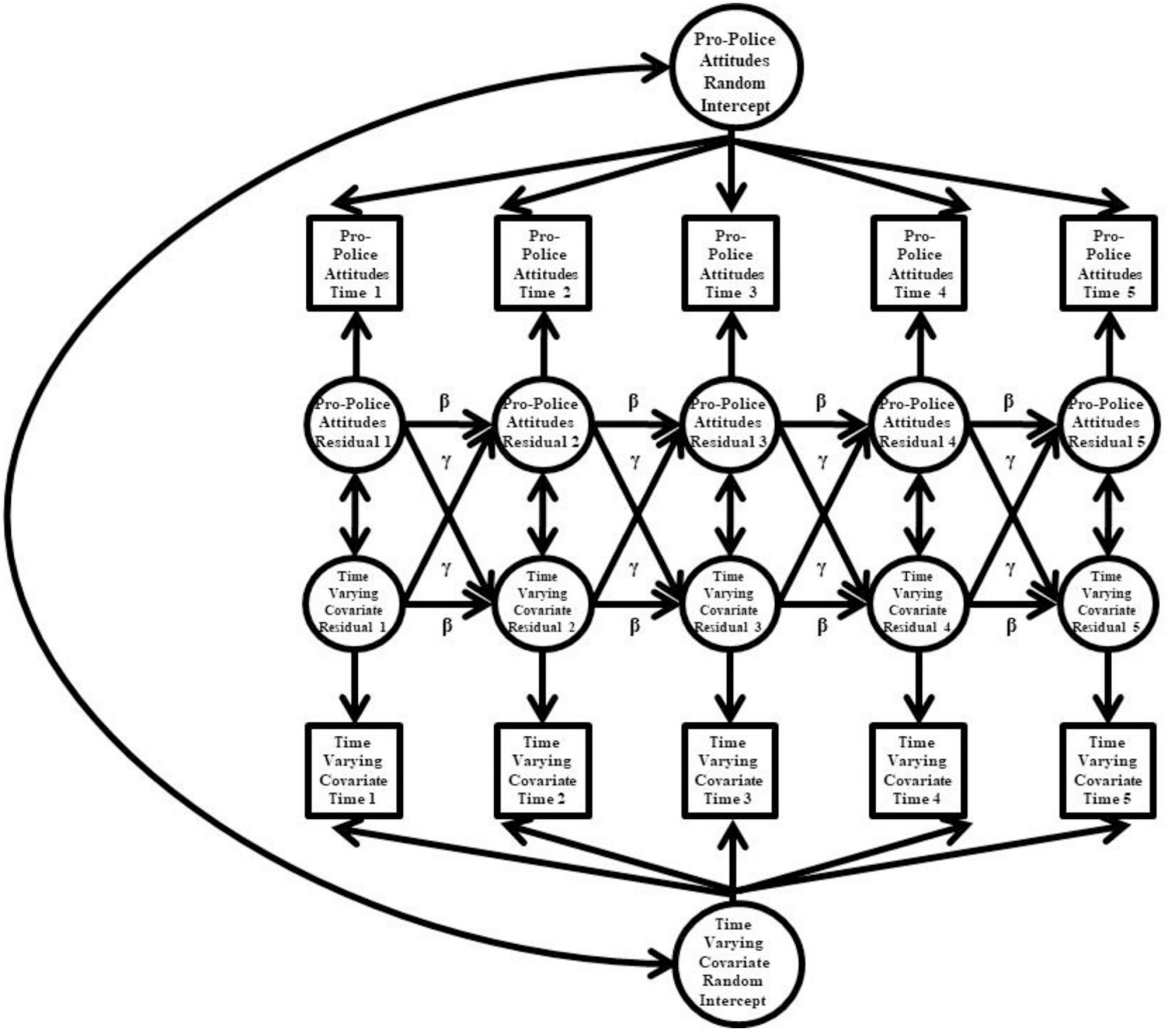
Random Intercept Cross-Lagged Panel Model (RI-CLPM). β = autoregressive path; γ = cross-lagged path. Variances and mean structure omitted from figure for clarity of presentation. All factor loadings were fixed to 1. All autoregressive paths were freely estimated.

Lastly, we fit a series of bivariate Random Intercept Cross Lagged Panel Model (RI-CLPM [80]) (see Fig 2) to examine associations between police attitudes and time-varying covariates. As with the RIAM, the random intercept factor accounts for between-person effects while within-person effects are represented by the residual structure, which captures time-point specific deviations from an individual’s model implied score. Separate random intercept factors were specified for police attitudes and one other time-varying covariate. Covariances were added between the random intercept factors, and residual factors associated with the same wave of assessment. Cross-lagged paths were also added from one variable’s residual factors to the subsequent residual factor of the other variable (γ in Fig 2). Although we fit the full RI-CLPM, we only report the correlations among the random intercept factors due to the limitations in detecting reliable within-person change already discussed (the absence of associations among residual factors in the model can bias estimates of the correlations among the random intercept factors).

The main analyses were conducted in Mplus version 8.4 using either WLSMV estimation (for the measurement models) or full information maximum likelihood estimation (for the latent growth and random intercept models) [81]. Confidence intervals were derived via non-parametric, percentile bootstrapping with 10,000 draws, which performs well under a variety of data conditions [82]. These analyses were also facilitated by the Mplus Automation Package [83] in *R* [84].

## Results

### Measurement model of police attitudes across waves

For the measurement model, all item parameters were initially freely estimated, all factor means were fixed to 0, and all factor variances were fixed to 1. This model had an adequate fit to the data (RMSEA = .070, SRMR = .092; CFI = .943; TLI = .939); factor loadings were large across items and waves (mean λ = .76, range = .63 to .91). To link the factors (i.e., place them on the same metric) so that scores could be validly compared over time [85], the factor loadings and thresholds of corresponding items were fixed to equality across the factors, and the factor means and variances for waves 2 through 5 were freely estimated. The constrained model also fit the data adequately (RMSEA = .059, SRMR = .094; CFI = .950; TLI = .956); factor loadings were large in magnitude across items and waves (mean λ = .76, range = .63 to .92), and factor reliability was high (mean ω = .91; mean *H* = .92 [86]). Factor scores from the constrained model were generated using maximum a posteriori (MAP) scoring [87].

### Mean-level trends in pro-police attitudes from summer 2020 to autumn 2021

There was a small increase in mean-levels of the factor scores for pro-police attitudes between summer 2020 and autumn 2021 (*d* = .24), with the majority of this increase occurring between summer and autumn 2020 (*d* = .18). As mentioned previously, the slope mean parameter from the LGC-SR model was significant (.12, 95% Confidence Interval [CI]: .08, 16), but the slope variance parameter was not (.00, 95% CI: .00, .00). Although we could not examine predictors of within-person change, preliminary analyses suggested that differences in mean-levels of pro-police attitudes from time 1 to time 5 might differ across race and political party affiliation. Therefore, we used summary statistics to test for differences in the means of pro-police attitudes at time 1 (summer 2020) and time 5 (autumn 2021) for the total sample and then for different racial groups and political parties.

**Fig 3:**
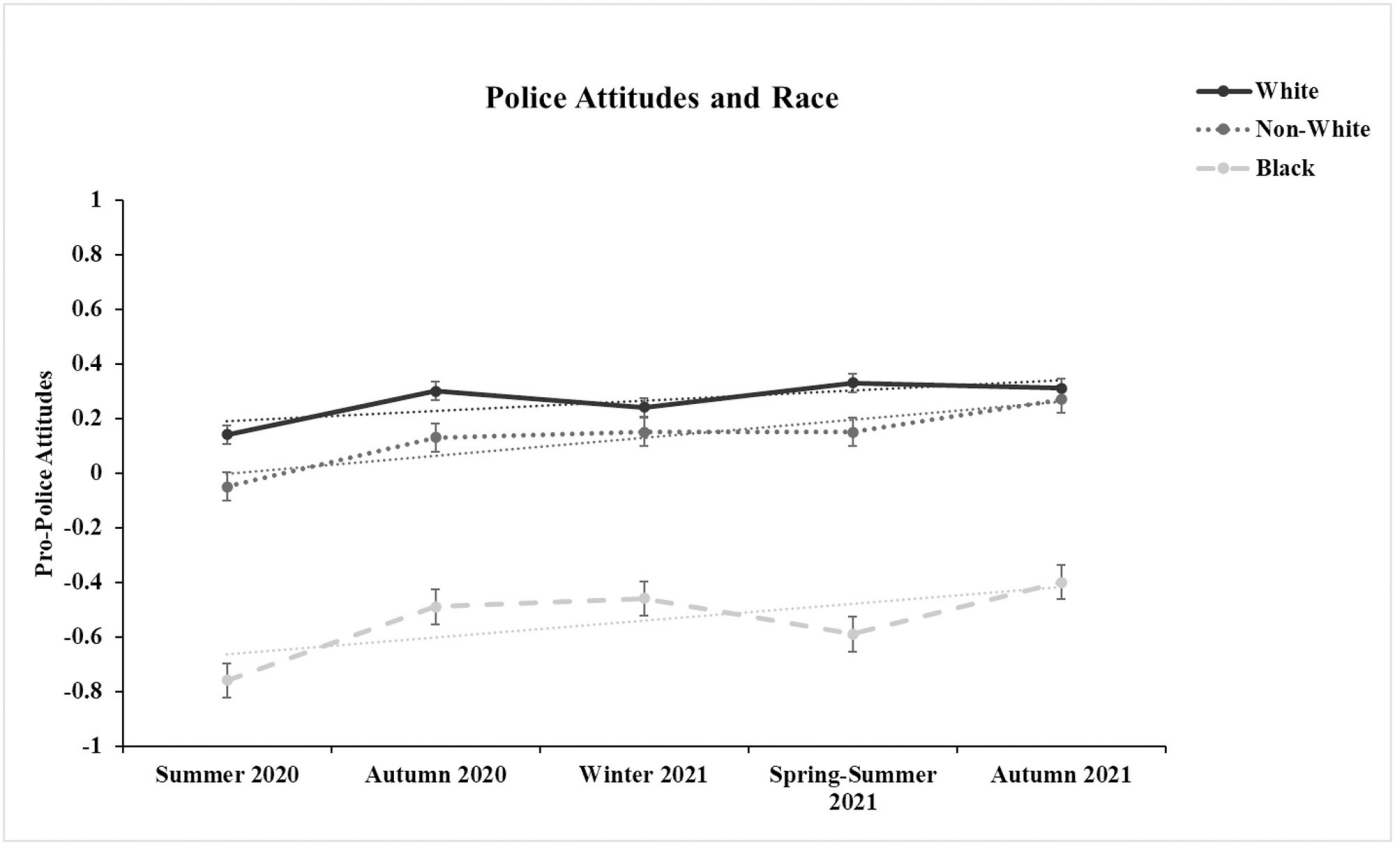
Pro-police attitudes are factor scores derived from the 5-factor confirmatory factor analysis model with items administered at a given time point loading solely on a single-factor at that timepoint. Number of participants across timepoints ranged from 761 to 785 for White, 135 to 145 for Black, and 84 to 115 for other non-White participants.

For the full sample, there was a significant increase in pro-police attitudes from time 1 to time 5 (*d* = .24, *t*[2043] = 5.42, *p* < .001), and this effect remained significant if the participants with data at both time points (*n* = 335) were excluded (*d* = .26, *t*(1373) = 4.86, *p* < .001). For racial groups, we found that Black (*d* = .51, *t*[278] = 4.28, *p* < .001) and other non-White participants (*d* = .40, *t*[197] = 2.78, *p* = .006) exhibited roughly twice the mean-level increase in pro-police attitudes compared to White participants (*d* = .20, *t*[1564] = 4.02, *p* < .001). Fig 3 illustrates the mean-level trends over time for the different racial groups. Results were consistent after removing participants with data at both time points: Black, *d* = .54, *t*(174) = 3.61, *p* < .001; other non-White, *d* = .44, *t*(143) = 2.58, *p* = .011; White, *d* = .23, *t*(1052) = 3.79, *p* < .001. We then converted the mean differences between time 1 to time 5 scores to correlations and tested whether the correlations were significantly different across racial groups. We found that the mean-level increase for Black participants (*r* = .25, 95% CI: .13, .36) was greater than White (*r* = .10, 95% CI: .05, .15) participants (*z* = 1.62, *p* = .052), but the mean-level increase for other non-White participants (*r* = .19, 95% CI: .06, .32) did not differ from either Black or White participants. At each time point, Black participants had much lower mean-levels of pro-police attitudes than White (mean *d* = -1.04) and other non-White participants (mean *d* = -.90), while the mean differences between White and other non-White participants was small (mean *d* = -.16). Descriptive statistics for pro-police attitudes broken down by racial group are reported in S1 Table.

**Fig 4:**
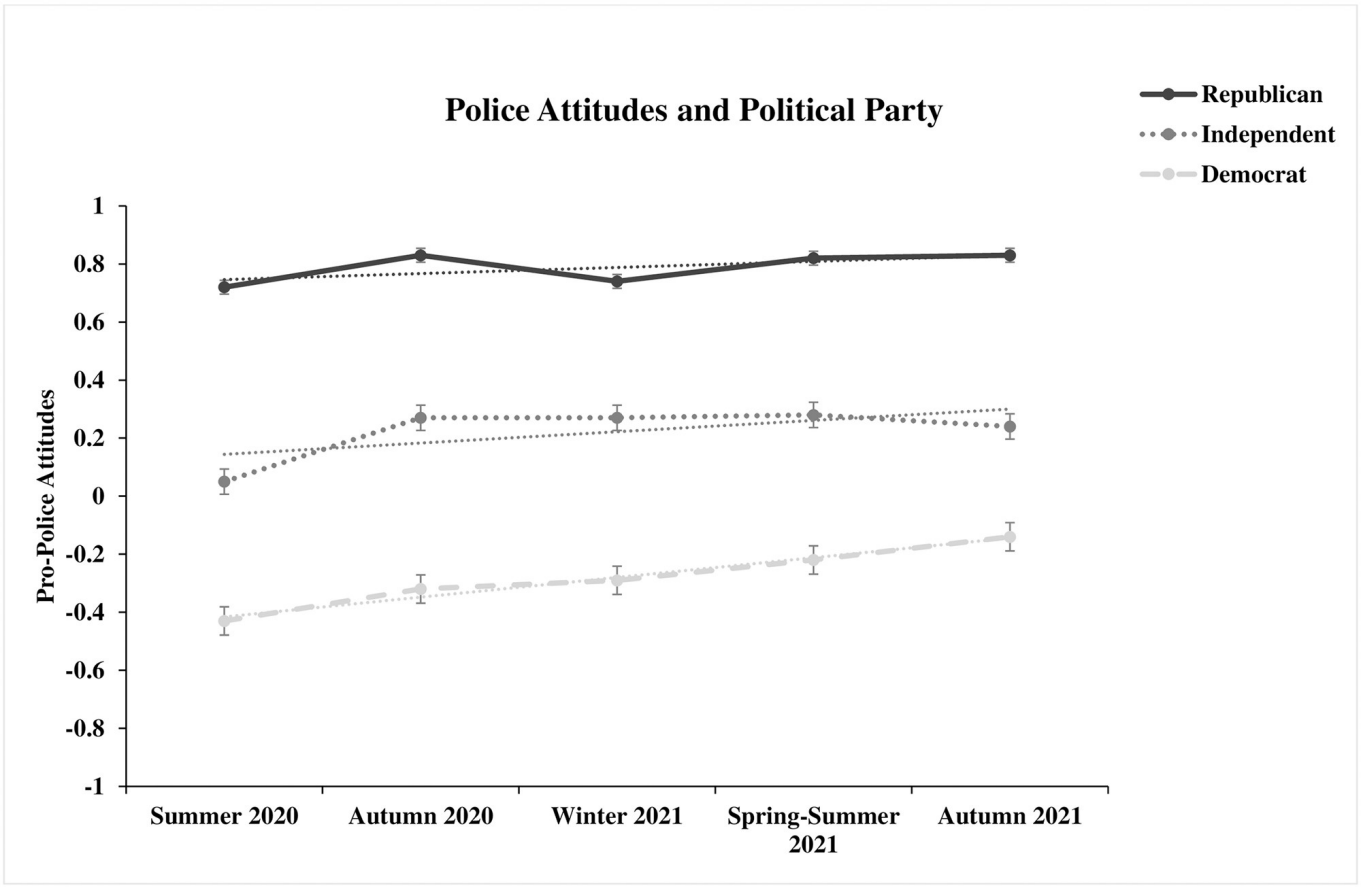
Pro-police attitudes are factor scores derived from the 5-factor confirmatory factor analysis model with items administered at a given time point loading solely on a single-factor at that timepoint. Number of participants across time points ranged from 411 to 448 for Democrats, 236 to 259 for Republicans, and 281 to 301 for independents or unaffiliated voters.

For political party affiliation, we found that Democrats (*d* = .40, *t*[875] = 5.98, *p* < .001) and independents (*d* = .24, *t*[589] = 2.88, *p* = .004) exhibited small-to-medium mean-level increases while Republicans did not exhibit a significant mean-level change in pro-police factor scores from time 1 to time 5 (*d* = .15, *t*[485] = 1.60, *p* = .110). Fig 4 illustrates the mean-level trends over time for different political party affiliations. Results were consistent after removing participants with data at both time points: Democrats, *d* = .49, *t*(565) = 5.80, *p* < .001; independents, *d* = .28, *t*(401) = 2.80, *p* = .005; Republicans, *d* = .12, *t*(337) = 1.09, *p* = .277. After converting the mean differences to correlations, we found that the mean-level increase for Democrats (*r* = .20, 95% CI: .13, .26) was greater than Republicans (*r* = .07, 95% CI: -.02, .16) (*z* = 1.58, *p* = .057), but the mean-level increase for independents (*r* = .12, 95% CI: .04, .20) did not differ from either Democrats or Republicans. At each time point, Republicans had higher (mean *d* = .72) and Democrats lower (mean *d* = -.66) mean-level scores of pro-police attitudes than independents, and Democrats had much lower mean pro-police attitudes than Republicans (mean *d* = -1.44). Descriptive statistics for pro-police attitudes broken down by political party affiliation are reported in S2 Table.

#### Associations between pro-police attitudes and demographics, political party, and personality traits

Next, we examined associations between the random intercept factor of pro-police attitudes and the time invariant covariates with correlations and regression coefficients reported in Table 3. Black versus White race was the strongest demographic correlate of higher pro-police attitudes, while older age, male sex, and higher income all had small associations with pro-police attitudes. In terms of political party, Democratic and Republican party affiliation was associated with lower and higher scores of pro-police attitudes, respectively. For personality traits, pro-police attitudes had small associations with low openness, low negative emotions, and conscientiousness, and a large association with RWA scores.

**Table 3:** Correlations, standardized regression coefficients, and 95% confidence intervals for associations between pro-police attitudes random intercept factor and time-invariant predictors.

Variable	*r*	Model 1	Model 2	Model 3	Model 4
**Demographics**					
Age	**.11 [.07, .15]**	**.18 [.14, .21]**	**.15 [.12, .19]**	**.11 [.07, .15]**	**.16 [.10, .22]**
Sex	**.10 [.06, .14]**	**.07 [.03, .11]**	**.06 [.02, .10]**	.05 [.01, .09]	.01 [-.04, .07]
non-White vs White race	-.03 [-.07, .01]	-.05 [-.09, -.01]	-.02 [-.06, .02]	-.03 [-.07, .00]	-.06 [-.11, -.01]
Black vs White race	**-.31 [-.34, -.27]**	**-.34 [-.38, -.30]**	**-.23 [-.27, -.19]**	**-.25 [-.28, -.21]**	**-.34 [-.40, -.28]**
Hispanic	.00 [-.03, .04]	-.02 [-.06, .02]	.00 [-.04, .04]	-.01 [-.04, .03]	-.05 [-.10, .00]
Income	**.09[.05, .12]**	**.07 [.03, .11]**	**.07 [.02, .11]**	.05 [.00, .09]	.06 [.00, .12]
Education	-.02 [-.06, .02]	**-.10 [-.14, -.06]**	**-.07 [-.11, -.03]**	**-.07 [-.11, -.03]**	.00 [-.06, .06]
**Political Party**					
Democrat vs Independent	**-.28 [-.32, -.24]**		**-.24 [-.28. -.20]**	**-.24 [-.28, -.20]**	**-.20 [-.26, -.14]**
Republican vs Independent	**.29 [.25, .33]**		**.26 [.22, .30]**	**.24 [.20, .29]**	**.11 [.04, .18]**
**Personality**					
Extraversion	.03 [-.02, .07]			.02 [-.02, .07]	-.04 [-.10, .02]
Agreeableness	-.02 [-.06, .02]			-.03 [-.07, .02]	-.06 [-.12, .00]
Conscientiousness	**.08 [.04, .12]**			.06 [.01, .11]	.05 [-.02, .12]
Negative Emotions	**-.10 [-.14, -.05]**			**-.10 [-.15, -.04]**	-.08 [-.16, -.01]
Openness	**-.17 [-.21, -.13]**			**-.16 [-.20, -.12]**	-.03 [-.08, .03]
Right Wing Authoritarianism	**.51 [.45, .56]**				**.43 [.36, .49]**
*R* ^2^		.136	.315	.348	.532

*Note*. Bold = *p* < .005. Sex 0 = female, 1 = male; non-White (excludes Black) = 1, White = 0; Black = 1, White = 0; non-Hispanic = 0, Hispanic = 1; Democrat = 1, Independent = 0; Republican = 1, Independent = 0. Confidence intervals were calculated using percentile bootstrapping with 10,000 random draws.

We then fit a series of models wherein we regressed the random intercept of pro-police attitudes on the demographic variables, political party, and personality traits in turn reported in Table 4. We began by including the demographic variables in model 1. After adjusting for their overlap, Black vs White race remained the strongest predictor of police attitudes followed by older age, while male sex, higher income, and now lower education had small unique associations with pro-police attitudes (*R*^2^ = .136, *p* < .001). In model 2, we added political party affiliation to the model along with demographic variables, which yielded a large increase in *R*^2^ (Δ*R*^2^ = .179, *p* < .001). The demographic variables retained their significant associations with pro-police attitudes, while Democratic and Republican party affiliation was associated with lower and higher pro-police attitudes, respectively. In model 3, we added scores on the BFI traits, which contributed to a significant increase in *R*^2^ (Δ*R*^2^ = .033, *p* < .001). Pro-police attitudes had small unique associations with low openness and low negative emotions over and above Black vs White race, political party affiliation, older age, and lower education. In model 4, we added RWA scores to the variables in model 3, and this resulted in a large increase in *R*^2^ (Δ*R*^2^ = .184, *p* < .001), with this final model accounting for 53.2% of the variance in pro-police attitudes. After adjusting for all the variables in the model, RWA scores had a large association with pro-police attitudes followed by Black vs White race, political party affiliation, and older age while the associations with scores on the BFI traits, sex, income, and education were no longer significant.

**Table 4:** Standardized regression coefficients and 95% confidence intervals for associations between random intercept factors for pro-police attitudes and time-varying covariates.

Criterion Variable	Unadjusted	Adjusted for Demographic covariates
**Political Attitudes**		
Race and immigration	**.66 [.62, .70]**	**.66 [.62, .70]**
Trump approval	**.66 [.62, .70]**	**.66 [.62, .71]**
Media bias	**.61 [.57, .65]**	**.62 [.58, .66]**
Pro-gun	**.42 [.38, .46]**	**.46 [.41, .50]**
Biden approval	**-.59 [-.64, -.54]**	**-.56 [-.61, -.51]**
Rigged 2020 election	**.59 [.51, .66]**	**.62 [.51, .69]**
Pro-insurrection	**.63 [.58, .67]**	**.64 [.60, .68]**
Trump idolatry	**.53 [.49, .58]**	**.53 [.49, .58]**
**Psychiatric Problems**		
Depression	**-.10 [-.16, -.05]**	**-.09 [-.15, -.04]**
Generalized anxiety disorder	**-.11 [-.17, -.04]**	**-.10 [-.19, -.05]**
Suicidal ideation and self-harm	-.02 [-.07, .01]	-.01 [-.06, .03]
Heavy drinking	.02 [-.03, .06]	.01 [-.04, .05]
Alcohol use problems	.00 [-.05, .04]	-.01 [-.06, .05]
Nicotine use	.01 [-.04, .04]	.02 [-.03, .06]
Antisocial behavior	-.04 [-.09, -.00]	-.03 [-.08, .02]
Intimate partner violence	.04 [.00, .07]	.07 [.02, .12]
**COVID-19**		
Approval of government restrictions	**-.49 [-.53, -.44]**	**-.51 [-.56, -.46]**
COVID-19 Safety behaviors	**-.36 [-.41, -.30]**	**-.38 [-.43, -.32]**
COVID-19 Worry	**-.27 [-.32, -.22]**	**-.26 [-.31, -.21]**
COVID-19 Skepticism	**.45 [.39, .50]**	**.49 [.44, .56]**
Anti-vax attitudes	**.22 [.15, .27]**	**.32 [.25, .37]**

*Note*. Bold = *p* < .005. Demographic covariates included age, sex, race, ethnicity, income, and education. Confidence intervals were calculated using percentile bootstrapping with 10,000 random draws.

### Political attitudes and pro-police attitudes

We then examined associations between the random intercept factors for pro-police attitudes and the time-varying covariates that included political attitudes, psychiatric problems, and COVID-19 variables. Pro-police attitudes had a large association with attitudes about race and immigration in the United States, higher approval of Donald Trump, and beliefs that the media is biased against conservatives. Pro-police attitudes also had slightly smaller but still medium-to-large associations with pro-gun attitudes, pro-insurrection attitudes, Trump idolatry, and stronger beliefs that the 2020 Presidential election was rigged in favor of Joe Biden. Pro-police attitudes also had a large association with low approval of President Biden. Adjusting for the demographic variables of age, sex, race, ethnicity, income, and education had little to no impact on the size of the associations between political attitudes and pro-police attitudes (mean β = .59 for both the unadjusted and adjusted associations with the political attitudes variables).

### Psychiatric problems and pro-police attitudes

Pro-police attitudes had small negative associations with depression and generalized anxiety disorder, and these associations remained significant after adjusting for the demographic variables. Pro-police attitudes were not associated with suicidal ideation and self-harm, and–contrary to predictions–were not associated with any of the substance use measures, antisocial behavior, or intimate partner violence (mean β = .02 and .03 for the unadjusted and adjusted associations, respectively).

### COVID-19 variables and pro-police attitudes

Pro-police attitudes had medium-to-large associations with less approval of government mandates and restrictions to mitigate the spread of COVID-19, less engagement in COVID-19 related safety behaviors, less worry about infection and negative health effects of COVID-19, and greater skepticism about the seriousness of COVID-19. Pro-police attitudes also had a small-to-medium association with more anti-vax beliefs. Adjusting for demographic variables had little to no impact on the size of the associations between pro-police attitudes and the COVID-19 variables. Because attitudes about COVID-19 are polarized across political parties, we also tested whether political party affiliation accounted for the association between pro-police attitudes and the COVID-19 variables. Adding political party affiliation to the adjusted model accounted for only 14% to 34% of the associations between pro-police attitudes and the COVID-19 variables, and pro-police attitudes continued to have significant associations with low approval of government restrictions (β = -.41, 95% CI: -.47, -.36), low engagement in COVID-19 related safety behaviors (β = -.28, 95% CI: -.34, -.22), less worry about the negative health effects of COVID-19 (β = -.19, 95% CI: -.24, -.14), greater skepticism about the seriousness of COVID-19 (β = .42, 95% CI: .36, .49), and more anti-vax attitudes (β = .21, 95% CI: .14, .27).

## Discussion

We examined change, stability, and several correlates of attitudes about police in the United States from June 2020 to October 2021, the period that followed the largest anti-police brutality protests in American history. We detected a small mean-level increase in pro-police attitudes over this period (*d* = .24). Most of this increase was observed from the summer to the autumn of 2020, indicating that to the extent pro-police attitudes declined at all, they recovered relatively quickly. Thereafter, mean-levels of pro-police attitudes remained relatively stable with high rank-order stability (6-month test-retest *r* > .85). Due to a lack of individual-level variability in the rate of change in pro-police attitudes over time, we were unable to identify predictors of change. We did, however, compare the means between the first (summer 2020) and last (autumn 2021) time points for key groups, and found some evidence for differences in mean-level change. Specifically, the mean increase in pro-police attitudes was greater for Black participants relative to White participants, and greater for Democrats relative to Republicans. The shift in scores among Black participants and Democrats was characterized by very low mean-levels at the first time point moving toward the grand mean over time, and the fact that White participants and Republicans had relatively high scores at the first time point and so exhibited only a small increase (i.e., scores were relatively stable) over time. Differences in mean-level change over time had only a minimal impact on the mean differences between these groups, as Black participants and Democrats had much lower pro-police attitudes at all time points relative to White participants and Republicans, respectively.

The largest demographic difference we observed was for race. Black participants had much lower mean-levels of pro-police attitudes than White and other non-White participants, and these differences were consistent across time despite the larger mean-level increase over time among Black participants relative to White participants. These results are consistent with prior findings of lower pro-police attitudes among Black Americans in general, as well as a larger increase in pro-police attitudes following a highly publicized event of police brutality [22, 88]. The pattern of a larger increase in pro-police attitudes among Black Americans, however, is clearly due to especially negative attitudes following a highly publicized act of police misconduct (i.e., a “floor” effect) that rebounds toward a historical mean rather than a meaningful improvement in police confidence among Black Americans following such events or a closing of the race gap in police attitudes. The low levels of pro-police attitudes among Black Americans are likely due to the historical use of police to enforce racially discriminatory laws and social norms emanating from a racial hierarchy of White dominance, as well as bias in policing and the enforcement of superficially race neutral laws that persists to the present day [89–92]. The finding that other non-White participants were much more similar to White participants in their attitudes toward police suggests that Black Americans in particular–rather than non-White Americans in general–have an especially contentious and complex relationship with police in the United States.

Older age was also a consistent though weaker correlate of pro-police attitudes, even after adjusting for other demographic variables, political party affiliation, and personality traits. This may be due to less formal interaction between older adults and police [93], as well as a greater identification with established authority figures and endorsement of the status quo of social institutions among older adults [94]. Male sex, higher income, and lower education had small associations with pro-police attitudes that were not significant after adjusting for personality, and Hispanic ethnicity was not associated with pro-police attitudes in any analysis.

Pro-police attitudes were polarized across political parties with high positive ratings among Republicans (random intercept T-score mean = 56.9, SD = 9.0) and low ratings among Democrats (mean = 45.4, SD = 8.4) with independents holding a middle position (mean = 50.8, SD = 9.5) between the two parties. Pro-police attitudes were also strongly associated with RWA scores that assess agreement with conservative and disagreement with liberal social and political attitudes and values in general. Pro-police attitudes had strong associations with conservative or right-wing attitudes about contemporary U.S. political issues including race and immigration, high approval of Donald Trump and low approval of President Biden, a belief that the American news media is biased against conservative viewpoints, and pro-gun attitudes, as well as beliefs that the 2020 presidential election was rigged for Democrats, sympathetic attitudes regarding the actions of insurrectionists who stormed the U.S. Capitol on January 6, 2021, and endorsement of Trump idolatry. These associations were virtually unchanged after adjusting for the demographic variables of age, sex, race, ethnicity, income, and education. At present then, pro-police attitudes seem to be firmly ensconced as a part of the conservative or right-wing political coalition in the United States.

Pro-police attitudes were largely unrelated to psychiatric problems, though we did detect small negative associations with depression, generalized anxiety, and BFI negative emotions scores. We predicted that substance use, antisocial behavior, and intimate partner violence would be associated with more negative attitudes toward police as these behaviors are associated with or defined by engaging in illegal activities and so increase the likelihood of official police contact and arrest. Surprisingly, none of the associations between these externalizing behaviors and pro-police attitudes were significant. This indicates that some people may be both relatively antisocial and pro-police or relatively prosocial and have negative attitudes about police. Also, the independence between pro-police attitudes and psychiatric problems suggests that pro-police attitudes may occupy a position in the right-wing ideological spectrum that is relatively normative as opposed to extreme (e.g., conspiracy theories about a rigged election or support for violent insurrection).

Pro-police attitudes also had significant, and in some cases large, associations with several COVID-19 related variables. The largest associations were with disapproval of government restrictions and mandates regarding mitigation strategies to reduce the spread of COVID-19 and skepticism about the seriousness of COVID-19, though pro-police attitudes also had robust associations with less engagement in safety behaviors, less worry about health risks of COVID-19, and more anti-vax attitudes, even after adjusting for demographic variables. Because attitudes about COVID-19 are polarized across political parties in the United States, we posited that political party affiliation would account for most of these associations (e.g., Republicans are more pro-police and skeptical about the seriousness of COVID-19). The associations between pro-police attitudes and the COVID-19 variables, however, remained relatively strong after adjusting for political party in addition to the demographic variables. Similar results were obtained when using RWA scores (a measure of non-specific conservative versus liberal political orientation) rather than political party. Why pro-police attitudes would continue to have incremental associations with COVID-19 variables after adjusting for a conservative or right-wing political orientation is unclear, and we have no alternative hypothesis at present. It may simply be that at the present historical moment the links between pro-police attitudes, right-wing political attitudes, and attitudes about COVID-19 are so strong that one cannot fully account for the association between the others.

### Police attitudes in 2020–2021

Generally, attitudes toward police have not altered substantially from their contemporary historical levels since the anti-police brutality protests that took place during the summer of 2020, but they have become more positive in comparison to the record low numbers during the protests [22]. Similar to other highly publicized acts of police misconduct, attitudes toward police fell sharply immediately following the broadcasted event but then slowly rose again, yielding few signs of sustained negative attitudes or political mobilization that is able to affect policy change [22, 57]. However, it will be necessary to track police attitudes for several more years to make comparisons with prior highly publicized events and determine the lasting impact–if any–of the George Floyd protests of 2020 on policing and attitudes about police in the United States.

Calls for policing reform have often followed periods of unrest, specifically related to systemic racism in law enforcement. However, most efforts to reform police departments tend to fade as public pressure decreases [95], reflecting the same trend that attitudes toward police follow after a high-profile event. Reform measures have ranged from improving small elements of policing such as stop and frisk procedures [96, 97] to the recent calls for the defunding of the police and diverting to other social services (i.e., reallocating public funds from police to other public health and safety services) [98]. Some departments implemented changes following the incidents and increased unrest, such as LAPD hiring more minority officers and greater oversight of the Los Angeles Police Commission following the Rodney King incident [60] or the national surge in police departments implementing body worn cameras following the civil unrest in Ferguson [99, 100]. However, many of these calls for reform have not been implemented effectively or worked out as policymakers hope. Stop and frisk reform in New York City, for example, has lowered the occurrences but has not stopped the procedure disproportionately affecting low-income residents and racial minorities [101]. Further, while police departments generally have become more diverse since the 1990s, many police departments are considerably more White than the communities they serve [102, 103], and about 90% of police chiefs and 82% of immediate supervisors in the United States are White [104]. A lack of diversity in police departments has major implications for police interactions and trust in communities [105, 106]. Finally, studies on body worn cameras show inconsistent and insignificant effects from their use by police departments on measurements of citizen or police behavior or even the public’s views on police [107, 108]. While instances of unrest have led to significant calls for reform, and sometimes to an attempt to change policies, many of these reforms have failed to meet their goals.

This is especially true when regarding calls to defund the police that occurred during the summer of 2020 but have since all but dropped off as a feasible policy position. In Minneapolis, where the defund the police movement first gained substantial traction, an initiative known as Yes 4 Minneapolis was put to a vote in November of 2021 [109]. The amendment’s goal was to effectively replace the Minneapolis Police Department with the Department of Public Safety, the first initiative of its kind that would follow the calls to defund the police [109]. Voters rejected the amendment, and, just a month later, the 2022 budget showed Minneapolis Police Department spending almost back at pre-protest levels [110]. This was not unique to Minneapolis either, as cities which tried or succeeded in lowering police budgets ended up reversing these goals in favor of the same, if not more, spending in their police departments from before the protests began [111]. This has been further emphasized by the focus of the Democratic party moving into the 2022 midterm elections. Reports of an increase in homicides and crime rates nationwide during the pandemic [112, 113] have spurred calls for change among both Republicans and Democrats, with many on both sides supporting an increase in law enforcement resources [114]. While some Democrats still advocate for defunding the police [115], many have attempted to reject this slogan amid Republican hopes to blame the increase in crime on the Democrats’ “soft-on-crime” policies [114–116]. During his 2022 State of the Union address, President Biden stated to bipartisan applause that “the answer is not to defund the police. It’s to fund the police” [117].

### Limitations and future directions

These results need to be interpreted in the context of several limitations. The first is that we do not have a measurement of police attitudes prior to the murder of George Floyd (May 26, 2020) and the initial protests which began the following day and spread to over 100 cities in the following 2 weeks (the first survey was in the field from June 9 to June 22, 2020). Consequently, we were unable to estimate the extent of the decline in pro-police attitudes (if any) nor were we able to determine if pro-police attitudes have returned to or exceeded their mean-levels prior to the summer of 2020. We did not detect significant variance in the slope parameter or rate of change in pro-police attitudes, precluding our ability to identify any predictors of change. This was likely due to the large proportion of participants who completed only a single assessment and so did not contribute to the variance of within-person change. The fact that we found racial group membership and political party affiliation was associated with differential mean-level change suggests that predictors of change exist, but that we were unable to identify them in this data set. Further, pro-police attitudes exhibited high rank-order stability between assessment waves that were only 3–4 months apart, which suggests that levels of absolute change may have been relatively small. However, our psychometric approach of using multiple items to conceptualize and measure police attitudes as a trait construct–rather than using single poll questions–provided greater information and precision in measuring individual’s attitudes about police, which might have contributed to greater stability in the scores. That is, attitudes about police may be more stable than single poll questions would suggest.

Though we conceptualized police attitudes as a trait construct within the psychological tradition, it is notable that police attitudes seem sensitive to highly publicized events that–while of broad interest–do not have a direct impact on most people (e.g., relative to a personal experience with police). This is in contrast to constructs such as personality traits (e.g., extraversion, neuroticism) which do not seem to be sensitive to highly publicized but distal events. Police attitudes also had strong associations with race and political party membership, which are socially constructed groups and generally not associated with personality and similar trait constructs. Therefore, while police attitudes can be measured as an individual differences construct, these attitudes might best be conceptualized as a hybrid sociological-psychological construct in terms of its determinants.

Despite these limitations, this paper provides important contributions to the current conversation surrounding attitudes toward police. By using measures formulated around current events, such as approval of Donald Trump and COVID-19 attitudes, we connect more common correlates of attitudes toward police to an updated and robust discussion of modern right-wing politics. Especially in considering the changes, or attempted changes, to policing in light of recent police brutality events such as the murder of George Floyd, our paper sheds light on specific areas of focus for policy considerations. Finally, these results validate a new measure of police attitudes, which includes an item related to the Black Lives Matter movement and provide thorough information on trends in police attitudes in the months following the largest mass protests against police brutality in American history.

For future directions, it will be important to continue to monitor mean-levels of pro-police attitudes, ideally in a sample with significant variability in the rate of change to identify predictors of change. It will also be important to continue to examine the correlates of police attitudes. At present, pro-police attitudes are strongly associated with right-wing political attitudes in the United States, but these associations could change if the policies or composition of political coalitions in the United States also changes. Also, events whether positive (e.g., police reforms) or negative (e.g., increase in crime, highly publicized incidents of police brutality) could also affect both the mean-levels and correlates of police attitudes. We believe the items and scales used in this report can be helpful in research on these topics.

## Supporting information

S1 Table Descriptive statistics and mean differences in pro-police attitudes across racial groups. (DOCX)

S2 Table Descriptive statistics and mean differences in pro-police attitudes across political party. (DOCX)
