# Supplementary material for: Attitudes about police and race in the United States 2020–2021: Mean-level trends and associations with political attitudes, psychiatric problems, and COVID-19 outcomes
Source: PLoS One. 2022 Jul 27;17(7):e0271954. doi: 10.1371/journal.pone.0271954 (PMC9328541; doi:10.1371/journal.pone.0271954)
Supplement: S1 Table — (DOCX) [file pone.0271954.s001.docx]

**Supplemental Table 1. Descriptive statistics and mean differences in pro-police attitudes across racial groups.**

| **Pro-Police Attitudes** | **Full Sample** | **1. White** | **2. Non-White** | **3. Black** | **1 vs 2** | **1 vs 3** | **2 vs 3** |
| --- | --- | --- | --- | --- | --- | --- | --- |
| **Summer 2020** |  |  |  |  |  |  |  |
| *M* | 0 | 0.14 | -0.05 | -0.76 | -0.22 | **-1.11** | **-0.90** |
| *SD* | 0.912 | 0.88 | 0.84 | 0.74 |  |  |  |
| *N* | 1008 | 785 | 84 | 139 |  |  |  |
| **Autumn 2020** |  |  |  |  |  |  |  |
| *M* | 0.17 | 0.30 | 0.13 | -0.49 | -0.19 | **-0.97** | **-0.78** |
| *SD* | 0.933 | 0.93 | 0.89 | 0.69 |  |  |  |
| *N* | 1004 | 766 | 93 | 145 |  |  |  |
| **Winter 2021** |  |  |  |  |  |  |  |
| *M* | 0.14 | 0.24 | 0.15 | -0.46 | -0.12 | **-0.89** | **-0.83** |
| *SD* | 0.846 | 0.83 | 0.73 | 0.74 |  |  |  |
| *N* | 985 | 761 | 88 | 135 |  |  |  |
| **Spring-Summer 2021** |  |  |  |  |  |  |  |
| *M* | 0.19 | 0.33 | 0.15 | -0.59 | -0.23 | **-1.25** | **-1.04** |
| *SD* | 0.846 | 0.81 | 0.77 | 0.64 |  |  |  |
| *N* | 1018 | 768 | 109 | 137 |  |  |  |
| **Autumn 2021** |  |  |  |  |  |  |  |
| *M* | 0.21 | 0.31 | 0.27 | -0.40 | -0.05 | **-0.97** | **-0.92** |
| *SD* | 0.808 | 0.79 | 0.78 | 0.67 |  |  |  |
| *N* | 1037 | 781 | 115 | 141 |  |  |  |
| **Time 5 - Time 1** | **0.24** | **0.20** | *0.40* | **0.51** |  |  |  |
| *r time1,time5* | .12 | .10 | .19 | .25 |  |  |  |
| 95% Confidence interval | .08, .16 | .05, .15 | .06, .32 | .13, .36 |  |  |  |

*Note.* Mean differences are Cohen’s *d.* Bold indicates *p* < .005; italics indicates *p* < .01.
