# Supplementary material for: Attitudes about police and race in the United States 2020–2021: Mean-level trends and associations with political attitudes, psychiatric problems, and COVID-19 outcomes
Source: PLoS One. 2022 Jul 27;17(7):e0271954. doi: 10.1371/journal.pone.0271954 (PMC9328541; doi:10.1371/journal.pone.0271954)
Supplement: S2 Table — (DOCX) [file pone.0271954.s002.docx]

**Supplemental Table 2. Descriptive statistics and mean differences in pro-police attitudes across political party.**

| **Pro-Police Attitudes** | **Full Sample** | **1. Democrat** | **2. Independent** | **3. Republican** | **1 vs 2** | **1 vs 3** | **2 vs 3** |
| --- | --- | --- | --- | --- | --- | --- | --- |
| **Summer 2020** |  |  |  |  |  |  |  |
| *M* | 0 | -0.43 | 0.05 | 0.72 | **0.60** | **1.49** | **0.83** |
| *SD* | 0.912 | 0.76 | 0.83 | 0.79 |  |  |  |
| *N* | 1008 | 429 | 301 | 243 |  |  |  |
| **Autumn 2020** |  |  |  |  |  |  |  |
| *M* | 0.17 | -0.32 | 0.27 | 0.83 | **0.72** | **1.46** | **0.66** |
| *SD* | 0.933 | 0.75 | 0.88 | 0.82 |  |  |  |
| *N* | 1004 | 411 | 294 | 259 |  |  |  |
| **Winter 2021** |  |  |  |  |  |  |  |
| *M* | 0.14 | -0.29 | 0.27 | 0.74 | **0.76** | **1.41** | **0.61** |
| *SD* | 0.846 | 0.70 | 0.78 | 0.76 |  |  |  |
| *N* | 985 | 419 | 281 | 236 |  |  |  |
| **Spring-Summer 2021** |  |  |  |  |  |  |  |
| *M* | 0.19 | -0.22 | 0.28 | 0.82 | **0.68** | **1.43** | **0.69** |
| *SD* | 0.846 | 0.68 | 0.79 | 0.77 |  |  |  |
| *N* | 1018 | 432 | 295 | 240 |  |  |  |
| **Autumn 2021** |  |  |  |  |  |  |  |
| *M* | 0.21 | -0.14 | 0.24 | 0.83 | **0.53** | **1.38** | **0.79** |
| *SD* | 0.808 | 0.68 | 0.77 | 0.73 |  |  |  |
| *N* | 1037 | 448 | 290 | 244 |  |  |  |
| **Time 5 - Time 1** | **0.24** | **0.40** | **0.24** | 0.15 |  |  |  |
| *r time1, time5* | .12 | .20 | .12 | .07 |  |  |  |
| 95% Confidence interval | .08, .16 | .13, .26 | .04, .20 | -.02, .16 |  |  |  |

*Note.* Mean differences are Cohen’s *d.* Bold indicates *p* < .005.
